# Properties of Central and Peripheral Concepts of Emotion in Japanese and Korean: An Examination Using a Multi-Dimensional Model

**DOI:** 10.3389/fpsyg.2022.825404

**Published:** 2022-02-15

**Authors:** Eun-Joo Park, Mariko Kikutani, Naoto Suzuki, Machiko Ikemoto, Jang-Han Lee

**Affiliations:** ^1^Faculty of Psychology, Doshisha University, Kyoto, Japan; ^2^Institute of Liberal Arts and Science, Kanazawa University, Ishikawa, Japan; ^3^Department of Psychology, Chung-Ang University, Seoul, South Korea

**Keywords:** emotion structure, emotion concepts, prototype perspective, multi-dimensional model, Japanese and Korean

## Abstract

The concept of emotion can be organized within a hypothetical space comprising a limited number of dimensions representing essential properties of emotion. The present study examined cultural influences on such conceptual structure by comparing the performance of emotion word classification between Japanese and Korean individuals. Two types of emotional words were used; central concepts, highly typical examples of emotion, and less typical peripheral concepts. Participants classified 30 words into groups based on conceptual similarity. MDS analyses revealed a three-dimensional structure with valence, social engagement, and arousal dimensions for both cultures, with the valence dimension being the most salient one. The Japanese prioritized the social engagement over the arousal while the Koreans showed sensitivities to the arousal dimension. Although the conceptual structure was similar for the two countries, the weight of importance among the three dimensions seems to be different, reflecting each culture’s values and communication styles.

## Introduction

Human emotional experience is highly complex. One of the approaches to decipher the complexity is to organize emotions in terms of a limited number of characteristics or dimensions, such as how different colors can be distinguished in terms of the variations of brightness, hue, and saturation (Fontaine et al., 2007). The multi-dimensional model of emotion concepts presumes that emotional characteristics vary along some fundamental dimensions, such as positivity, and the strengths of these characteristics determine the position of each concept within the model. A crucial mission of research with this approach is to identify how many underlying dimensions there are and what properties they represent. The present research follows this line of investigation and focuses on two issues: the cultural influence on the dimensional structure of emotion and distinctions of prototypical and less prototypical emotions.

In order to identify underlying dimensions, inter-correlations among different emotions are measured through people’s perception and classification of emotion labels (Russell et al., 1989; Västfjäll et al., 2002; Yik and Russell, 2003), facial expressions (Cacioppo et al., 1986; Johnsen et al., 1995), and voices (Laukka et al., 2005; Szameitat et al., 2011). Important dimensions are then revealed through statistical techniques such as multidimensional scaling (MDS) and factor analysis (for a review, see Russell, 1980). Although researchers have been vigorously debating what the fundamental properties of emotion would be for the last few decades, a two-dimensional model with valence and arousal properties seems to be very prominent (see details in Bliss-Moreau et al., 2020). The valence dimension represents the degree of positivity and negativity of an emotional experience. Valence is thought to be a basic property of emotion, and this can be observed in self-reported experiences as well as in virtually all instrument-based measures of emotions (Russell and Barrett, 1999; Barrett, 2005; Barrett and Bliss-Moreau, 2009). The arousal dimension indicates how active, alert, or prepared the experiencer is while feeling the emotion. This dimension may be related to the excitatory state of neurons or the propensity of neurons to discharge when the emotion is experienced (Heilman, 2000).

However, the idea that the fundamental properties of emotions can be reduced to just two dimensions has been criticized for its over simplicity (Trnka et al., 2016), and several researchers have suggested few extras. In fact, some of the oldest research on this matter suggested a three-dimensional structure with an additional characteristic to valence and arousal (Wundt, 1896). The feature of the third dimension differs across research. Examples are relaxation-tension (Wundt, 1896), attention-rejection (Schlosberg, 1954), and control-impulse (Osgood, 1966). More recent research has proposed four-dimensional models with valence, arousal, control, and novelty (Fontaine et al., 2007; Fontaine, 2013; Fontaine and Scherer, 2013). A dimension of emotional engagement, which concerns whether the emotional experience fosters individuals’ independent sense of self or interdependent sense of self, has also been identified (Kgantsi et al., 2015). These prominent dimensions are proposed as important characteristics for people to distinguish different emotions. However, whether these are culturally universal or not is still unclear.

The valence/arousal two-dimensional structure has been found in many different cultural or language groups (Russell, 1980; Russell et al., 1989; Västfjäll et al., 2002; Yik and Russell, 2003; Posner et al., 2005; Jackson et al., 2019), but this could be the result of over-reducing dimensions (Trnka et al., 2016). It is highly possible that some culturally specific dimensions exist (Kuppens et al., 2006) or, even within the same dimensional structure, the order of importance among the dimensions could vary across cultures. Therefore, the present research compares the multi-dimensional structure of emotion between two cultures: Japan and Korea.

The reason behind focusing on Japan and Korea is the authors’ previous investigation on emotion prototypes for these two cultures (Park et al., 2018). Rosch (1978) proposed that all our learned concepts (e.g., colors and shapes) are organized as a collection of non-arbitrary semantic categories that develop around perceptually salient “natural prototypes.” Within each category (e.g., birds), more typical exemplars (e.g., a robin) are located at the center, whereas less typical exemplars (e.g., a penguin) are located at the periphery of these items. Fehr and Russell (1984) found that the prototype perspective also applies to emotion, and different emotional words can be classified as central concepts with high typicality or peripheral concepts with a lower level of typicality. The central concepts represent well-defined, more basic emotions, including happiness, anger, and sadness, whereas the peripheral concept words represent more complex and less basic emotions such as boredom, respect, and awe.

Park et al. (2018) replicated the research by Fehr and Russell (1984) with Japanese and Korean individuals and found a similar central/peripheral division for their emotion concepts. They first asked their participants to report words that are examples of emotion and defined the five most frequently reported words as central concepts. Three out of five central concepts (sad, delighted and enjoyable) were equivalent between the two cultures, while the other two words did not match. Overall, most central concepts found in the two countries were similar to the central concepts reported by the English-speaking participants in Fehr and Russell (1984). Words that the participants mentioned much less frequently were regarded as peripheral concepts of emotion, and five randomly chosen words from the set were used as representatives. Using the five central and five peripheral words, Park et al. (2018) investigated the functions of the two types of concepts and how Japanese and Korean people classify them. It is revealed that central concept words are not only better examples of emotion but also able to serve as a better substitute for the word “emotion” in sentences compared to peripheral concepts. The research demonstrated that Japanese and Korean participants distinguished these two types of concepts consistently across four studies, although the distinction was found to be more ambiguous for Koreans than for Japanese.

The present research has two purposes. The first is to reveal the multi-dimensional structure of emotion concepts for Japanese and Korean individuals, and compare them. Considering the research in other countries, dimensions of valence and arousal are highly expected to be found (Russell, 1980; Russell et al., 1989; Västfjäll et al., 2002; Yik and Russell, 2003; Posner et al., 2005; Jackson et al., 2019), but there might be some additional dimensions. Although some studies have investigated the Japanese and Korean structures separately, no systematic comparison across the two cultures has been made (see more details in the following section of the introduction). The second purpose of this research is to examine the properties of central and peripheral concepts. The previous research by Fehr and Russell (1984) and by Park et al. (2018) did not determine which properties of emotion are used to distinguish central and peripheral concepts. The latter research neither established whether Japanese and Koreans use similar criteria for the distinction. If the locations of central and peripheral concepts on the multi-dimensional space show a systematic order (e.g., they tend to be located along different dimensions), it is possible to infer crucial properties distinguishing these two types of concepts. In the present study, participants viewed 15 central and 15 peripheral words and classified them into some groups according to semantic similarity. The performance was analyzed using MDS to identify the underlying dimensions.

The present study used the central and peripheral words chosen for each culture. This enables the inclusion of culturally specific emotion words, such as Korean’s *han* (resentment), which are often excluded to ensure stimulus equivalence across different cultures. Overlooking or excluding culturally unique words because they are difficult to match between cultures will obscure cultural variations of dimensional structure. In addition, this treatment is likely to prevent biased familiarity for certain words favoring one culture or the other. Although the word sets in Japanese and Korean are not perfectly matched in the present study, there are also quite a few words with equivalent meanings. Thus, the current stimuli are suited to observe both culturally invariant and culturally variant aspects of the structure.

The word classification method used in the present research is also designed to reduce bias. There are various ways to assess how people perceive emotion. One of the most common methods is to rate each emotional stimulus according to some predetermined characteristics (e.g., rating how positive or negative the emotion is). Those characteristics are often related to dimensions which researchers expect to find. In fact, there are some well-established scales related to the two-dimensional model (Larsen and Diener, 1992; Feldman Barrett and Russell, 1998), and either direct translations or adopted versions of them have been used in various cross-cultural studies (Västfjäll et al., 2002; Yik and Russell, 2003). This procedure, however, directs participants’ attention to specific characteristics of emotion, and thus, any possible subtle cultural variations on emotion perception can be obscured. The GRID instrument created by Scherer (2005) also asks participants to rate each emotion in terms of some characteristics, but this scale covers more diverse features (144 of them) beyond valence and arousal. Four dimensions have been found by research using this instrument (e.g., Fontaine et al., 2007). Nevertheless, it is unavoidable for these rating tasks to give participants some indications about how they are supposed to judge emotions. In order to avoid this, the present research used a simple grouping task. Participants were presented with 30 emotion words at once and asked to divide them into a certain number of groups based on similarity. No instruction was given about possible emotional features available to use for creating groups. A similar procedure was used in the study by Fontaine and colleagues, which compared the emotion structure of Indonesian and Dutch individuals (Fontaine et al., 2002). They found three dimensions of valence, arousal, and control for both countries (valence and control were called evaluation and dominance in their study).

As a final part of this introduction, some cultural differences in emotion processing between Japanese and Koreans are highlighted. Both countries are in East Asia, and they are categorized as a collectivistic or interdependent cultural group, as opposed to an individualistic or independent group including most Western countries (Markus and Kitayama, 1991). However, recent research has pointed to some significant differences in emotion processing between Japanese and Koreans. For example, some facial expressions signifying the same emotion are reported to be slightly different (Daibo, 2007; Wang and Maiya, 2008). Japanese and Koreans also differ in terms of their functions of emotional expressions. On the one hand, Japanese people have a strong tendency to suppress their true feelings and avoid expressing them to others to prevent confrontations or conflicts. It is because the emotional expression of Japanese is harmony oriented (Zhao, 2002; Takatsuki, 2008). On the other hand, Korean individuals prefer to express their emotions to others even when it causes conflicts because they value the process of understanding and empathizing each other through arguments (Lee and Matsumoto, 2011). Thus, the behaviors of Koreans are more emotion oriented (Choi, 1993). Such tendencies are reflected in expressions of anger in the two languages which reference body parts. Many Japanese phrases relating to anger include the word for “stomach”, an internal part of the body, whereas the most frequently used body parts for Korean angry expressions are “eyes” (M. O. Lee, 2006). These cultural differences may influence the conceptual structure of emotion.

Regarding previous research on the dimensional structure, the standard two-dimensional model has been found for Japanese (Honma, 2014). A three-dimensional structure with energy, tension, and hedonic tone is also suggested (Joh, 2009). Korean structures have been more inconsistent across studies. In addition to the standard two-dimensional model (Yik et al., 2003), other two-dimensional combinations have been proposed, such as valence and activity-passivity (Han, 2000), or valence and self-other focused attention (Park and Min, 2005). Three-dimensional structures are also suggested, including a model with valence, activity-passivity, and external-internal direction (Lee and Lee, 1990), one with valence, arousal, and relaxation-tension (Kang and Han, 1994), and another with valence, arousal, and self-other focused attention (Lee et al., 2008). The presence of the arousal dimension is inconsistent among Korean models. This might be because Korean emotions are generally high in arousal level; in other words, there are insufficient variations of arousal level among different emotions. It has been found that the level of arousal for neutral and positive emotions is generally higher for Koreans than for Japanese (Kuppens et al., 2006; Uchida and Kitayama, 2009). Compared with American individuals, Park and Park (2009) report that Koreans show more highly aroused reactions to the International Affective Picture System (IAPS). Thus, the arousal dimension might be a key aspect of differences between Japanese and Korean conceptual structures.

## Materials and Methods

### Participants

All participants were undergraduate students. They were 63 Japanese (16 men and 47 women, Mean age = 20.08, SD = 0.94) at Doshisha University, and 64 Koreans (20 men and 44 women, Mean age = 22.71, SD = 0.27) at the Chung-Ang University. The present research design is based on Russell (1980) study, having 34 participants and 28 emotion words. The present research aimed to double the sample size from that study to improve the reliability of the analyses, which resulted in targeting approximately 65 participants in each cultural group. These sample sizes also meet the criterion suggested by Rodgers (1991) that it should be twice as large as the number of items participants make judgments about in the experiment. The present study used 30 words for each culture and thus, the required participants according to this criterion would be 60. All participants were treated in accordance with the APA ethical guideline and the treatment was approved by the ethical committee of Doshisha University. All participants gave written informed consent.

### Materials

Thirty Japanese and 30 Korean words selected from Park et al. (2018, Study 1) were used. The 15 most frequently reported words in that study were chosen as the central concept words for each language. Another set of 15 words was randomly selected from the rest of the list as the peripheral concept words. The set of central and peripheral words in Japanese and Korean were not matched, and the grammatical functions of the selected words were also varied. All the words used in this experiment are reported in Tables 1, 2 in the result section. Each word was written in 36 points MS Gothic font and printed on a card measuring 85 mm × 30 mm.

**TABLE 1:** MDS scores of emotion words in Japanese.

Words (English translation)	Words in Japanese	Weighted euclidean dimensions		
		1	2	3
**Painful**	***tsurai***	**0.97**	**1.37**	**–0.13**
**Agony**	***kurushii***	**1.16**	**0.97**	**–0.20**
**Sad**	***kanashii***	**0.33**	**0.91**	**0.22**
**Lonely**	***sabishii***	**0.33**	**1.30**	**–0.04**
**Exhausted**	***shindoi***	**0.85**	**0.71**	**1.57**
Languorous	*darui*	1.16	0.40	1.42
Tired	*tsukare*	0.70	**−−**0.09	1.81
Bored	*tsumaranai*	1.31	**−−**0.44	1.10
Cry	*naku*	0.17	0.28	0.66
**Fearful**	***kowai***	**0.26**	**0.10**	**–0.72**
Mortification	*kuyashii*	1.29	**−−**0.40	**−−**0.38
**Angry**	***ikari***	**0.46**	**–0.38**	**–1.73**
**Irritation (1)**	***iraira***	**0.53**	**–0.89**	**–1.33**
Resentment	*uramu*	1.10	**−−**0.53	**−−**1.49
**Hate**	***kirai***	**0.92**	**0.43**	**–1.67**
Surprised	*odoroki*	**−−**0.59	**−−**1.18	0.34
Excitement (2)	*kofun*	**−−**0.67	**−−**1.20	**−−**0.75
Nervous	*kincho*	0.02	**−−**1.71	0.37
Ashamed	*hazukashii*	0.42	**−−**1.83	0.99
Impatient	*aseri*	0.60	**−−**1.84	**−−**0.37
Irritation (2)	*modokashii*	0.86	**−−**1.26	0.34
**Enjoyable**	***tanoshii***	**–1.45**	**0.06**	**–1.30**
**Funny**	***omoshiroi***	**–1.12**	**0.13**	**–0.84**
Excitement (1)	*wakuwaku*	**−−**1.76	**−−**0.32	**−−**0.27
**Delighted**	***ureshii***	**–1.33**	**1.18**	**–1.09**
Kind	*yasashii*	**−−**1.47	0.33	1.33
Calm	*ochitsuku*	**−−**1.15	**−−**0.38	1.24
**Glad**	***yorokobi***	**–1.55**	**1.20**	**0.21**
**Like**	***suki***	**–1.24**	**1.76**	**–0.28**
**Happy**	***shiawase***	**–1.09**	**1.33**	**0.95**

**FIGURE 1:**
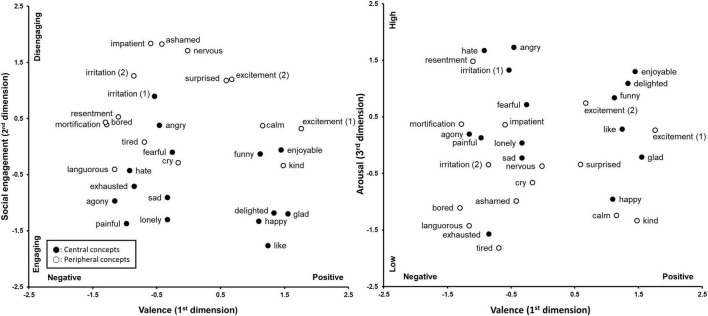
The three-dimensional structure of central and peripheral concepts in Japanese. The data is flipped 180 degrees vertically and horizontally from the original figure.

*The central concepts are in bold type and they are ordered by their typicality found in Park et al. (2018). The three-dimensional coordinates on this table were used to plot Figure 1. The original Japanese and Korean words varied in their parts of speech and thus, the English translations have been chosen to describe the meaning of the original words most precisely rather than strictly reflecting the part of speech of the original words.*

**TABLE 2:** MDS scores of emotion words in Korean.

Words (English translation)	Words in Korean	Dimensions		
		1	2	3
**Angry**	***hwanan***	**–0.82**	**1.02**	**1.49**
**Rage**	***bunno***	**–0.71**	**0.64**	**1.73**
Agony	*goeroun*	**−−**0.42	**−−**0.24	0.95
Resentment	*han*	**–1.12**	**−−**0.46	1.44
Hard	*himdeum*	**−−**1.48	0.00	0.24
**Irritation**	***ja-jeungnanun***	**-1.22**	**1.40**	**0.63**
Annoyed	*gwichaneun*	**−−**0.78	1.81	**−−**0.22
Cry	*ur-eum*	**−−**0.66	**−−**1.49	**−−**0.07
**Sad**	***sulpun***	**–0.84**	**–1.47**	**0.77**
**Lonely**	***oaeroun***	**–1.16**	**–0.89**	**–1.12**
**Depressed**	***uulhan***	**–1.46**	**-0.96**	**–0.16**
Mortification	*eok-ulhan*	**−−**0.58	**−−**0.30	**−−**0.34
Ashamed	*bukkuereoun*	0.15	**−−**0.05	**−−**0.90
**Surprised**	***nollaun***	**0.79**	**0.56**	**–1.48**
Nervous	*kinjang*	**−−**0.16	1.56	**−−**1.13
Impatient	*chojohan*	**−−**0.54	1.22	**−−**1.50
Fearful	*duryoeun*	**−−**1.03	0.78	**−−**0.80
Bored	*jiruhan*	**−−**0.89	0.01	**−−**2.01
Lively	*hwalgichan*	1.37	**−−**1.56	0.01
**Funny**	***utgi-neun***	**0.87**	**–1.51**	**-0.73**
Comfortable	*pyeon-anhan*	0.54	**−−**0.99	0.05
**Exult**	***sinnaneun***	**1.28**	**–0.82**	**–1.24**
**Enjoyable**	***julgoeun***	**1.02**	**–0.70**	**1.26**
**Delighted**	***kippun***	**1.25**	**–1.17**	**1.16**
**Love**	***sarang***	**0.96**	**0.46**	**1.48**
Excitement	*heungbun*	0.41	0.80	0.10
**Heart fluttering**	***seol-lem***	**1.06**	**1.02**	**0.62**
Pounding	*dugeungeorinun*	1.03	1.37	**−−**0.18
**Like**	***joa-haneun***	**1.56**	**0.13**	**–0.54**
**Happy**	***haengbok***	**1.57**	**–0.17**	**0.50**

**FIGURE 2:**
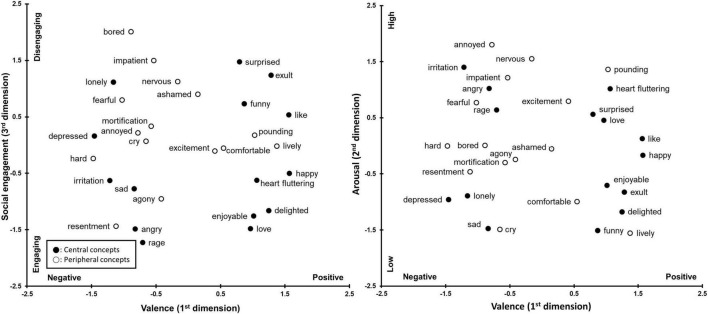
The three-dimensional structure of central and peripheral concepts in Korean.

*The central concepts are in bold type and they are ordered by their typicality found in Park et al. (2018). The three-dimensional coordinates on this table were used to plot Figure 2.*

### Procedure

The present study employed a procedure used by Russell (1980). Participants were tested individually. They were initially presented with the 30 cards placed on a desk and asked to divide them into a specific number of groups according to semantic associations. Once participants finished classifying the words, the experimenter recorded the groupings and then shuffled the cards for the next trial. There were four trials in which participants were asked to make three, six, eleven, and fourteen groups. Half of the participants were asked to make the number of groups in ascending order. The order was reversed for the rest. For the grouping, Russell (1980) used four, seven, ten, and thirteen groups. The idea behind the original grouping was to use two odd numbered and two even numbered groups, with one being small and one being large (the four was the small even group and the ten was the large even group). The detail is explained in Fehr and Russell (1984) as well as Armstrong et al. (1983). Since the present study used two more words than Russell’s study, the range of the groupings was stretched a little more while maintaining to use the two even and two odd numbered groups, which resulted in the three, six, eleven and fourteen groupings.

## Results

### Data Coding

The data for the two languages were treated separately. Since 30 words were used, the number of possible two-word combinations can be calculated from the following formula [(30 × 29)/2] = 435. Each of the 435 pairs was given a similarity score every time the two words were classified together. The score for each trial was set as identical to the total number of classified groups; so that three points were given to a pair classified together when participants were asked to sort the whole word set into three groups. The score of six, eleven, and fourteen points were given for the rest of the trials accordingly. All pairs also received 1 point regardless of the classification. Therefore, the pairs classified into the same group in all four trials obtained the maximum score of 35, while the pairs which were never classified together obtained the minimum score of 1. This coding was performed for the data from every individual participant.

### Multidimensional Scaling Analyses

The similarity scores for the 435-word pairs were transformed to a 30-words × 30-words matrix for each participant. These matrices were submitted to MDS analyses to show the relative spacing of the 30 words and their positions in a multi-dimensional space. PROXSCAL in SPSS version 26 was used for the analyses. PROXSCAL was selected because it weighs the similarity and dissimilarity of the ratings equally. This enables the resulted model to reflect conceptual distance among various emotions in such a way that emotions mapped close to each other are more similar than those mapped further apart (Bliss-Moreau et al., 2020).

In the analysis, the dependent variable was treated as an ordinal scale and thus, the proximity transformation of the model is set to ordinal. The current data consisted of a substantial number of pairwise similarity assessments, and this could bias the algorithm, so the “untie tied observation” function was used to resolve this issue. The weighted Euclidean model was employed to take into account individual differences among the responses, such as clustering of ratings within participant, and the importance of given dimension for each participant. The overall solution of the weighted Euclidean model is drawn by averaging out such individual differences, and thus the final solution cannot be rotated. The model also computes the importance of each dimension for each participant as dimension weights. Since this study used a different word set for Japanese and Korean languages, the similarity ratings obtained by the two groups of people must reflect their own cultural background and individual variations. Therefore, the weighted Euclidean procedure was used to reveal the structure for each of the two cultures in detail. The model’s shape was the lower triangular matrix, and its proximity was similarity based. The initial configuration of multidimensional scaling for this analysis was set to Torgerson. PROXSCAL procedure in SPSS repeatedly calculates the stress by adding one dimension at a time until its value falls below 0.01. The stress value would diminish gradually as more dimensions are added, but the most interpretable solution is determined as the point where an additional dimension ceases to reduce stress value noticeably. The Kruskal’s stress-1 value of a model indicates the goodness of fit as follows: 0.20 is poor, 0.10 is fair, 0.05 or below is good to excellent (Kruskal, 1964). In the present result, a three-dimensional solution was accepted for both countries because stress values of the models constantly dropped until the third dimension was added, but no noticeable reduction was observed after that (see Supplementary Material for the scree plot of this analysis). For the Japanese model, the RSQ value was 0.99 and Kruskal’s stress-1 value was 0.11. For the Korean model, the RSQ value was 0.99 and Kruskal’s stress-1 value was 0.12. These values meet the criteria of a good fit model (Hair et al., 2006). Tables 1, 2 show MDS sores of emotion words in Japanese and Korean. The central concepts are written in bold text.

The multi-dimensional structure is plotted on Figures 1, 2, in which central concepts are black circles. The figures represent original positions of the emotion concepts revealed in the MDS, and no rotation was performed. However, Figure 1 is flipped 180 degrees horizontally as well as vertically from the original figure for Japanese data. This was done to match the directions of the axis between Figures 1, 2.

In the Japanese structure, the first dimension clearly indicated valence, with positive emotions located on the right side and negative emotions on the left side. On the second dimension, emotions such as “nervous,” “impatient,” and “ashamed” were around the top, while “like,” “happy,” “painful,” and “lonely” were at the lower end. This dimension is identified as social engagement. The characteristic of this dimension concerns whether the emotion is related to the experience of independent sense of self (regarding self as an entity that is independent from social relationships) or interdependent sense of self (regarding self as being embedded in social relationships with others) (Markus and Kitayama, 1991; Kgantsi et al., 2015). Socially disengaged emotions make the independent-self salient, thus they are often experienced when the sense of autonomy is threatened (e.g., anger, surprise, nervous) or enhanced (e.g., pride). Socially engaging emotions heighten the interdependent sense of self and thus, those emotions often lead individuals to increase social interactions. Sadness and happiness are good examples of those. Sadness often works as a signal to draw others closer for help and support (Clark and Taraban, 1991; Van Kleef et al., 2010; Gray et al., 2011), and happiness and other positive emotions such as delighted or glad facilitate interpersonal cooperation by being shared among people (Van Kleef et al., 2010). The locations of Japanese emotion along the vertical axes of Figure 1 (left) appear to fit the interpretation of this dimension. However, not all the emotions were perfectly placed in accordance with the socially engagement characteristic. The emotion of ashamed (or shame), which is generally classified as a socially engaging emotion (Kitayama et al., 2000, 2006; Kgantsi et al., 2015), was on the socially disengaging side in the Figure 1. This issue is discussed later.

The social engagement dimension appears to be corresponding to the distinction between central and peripheral concepts for the Japanese. There is a clear tendency that socially engaging emotions are regarded as central concepts, and socially disengaging emotions are regarded as peripheral concepts. This means that Japanese are sensitive to whether the emotion is socially engaged or not, and regard socially engaging ones as highly typical examples of emotion.

The third dimension for the Japanese data was interpreted as arousal. Emotions such as “angry,” “hate,” “irritation,” and “excitement” have high arousal level and are located at the top end of the figure, while low arousal emotions such as “tired,” “calm,” “exhausted,” and “languorous” were at the bottom.

In the Korean structure, the first dimension was clearly the valence dimension, and the second was likely to be arousal, followed by the social engagement dimension. The arousal dimension is readily interpretable because highly aroused emotions such as “angry,” “nervous” and “pounding” were locating at the high end of the figure, while low arousal emotions such as “depressed” and “sad” are at the lower end. However, some low arousal emotions in the Korean structure were a little puzzling because emotions with a supposedly high arousal level, such as “lively” and “exult,” are located at the lower end of the axis in Figure 2. It is most likely due to the Korean word set’s lack of extremely low arousal emotions (e.g., tired and languorous). It is also noticeable that few words with mid-to high-arousal levels in the Japanese structure (e.g., sad, cry, funny, resentment, and enjoyable) were located much lower in the Korean structure. This suggests that the Korean arousal dimension could be extended toward the bottom of Figure 2 to capture even lower arousal emotions (which were not used in the present study). In other words, the arousal dimension in Figure 2 covers a narrower range of arousal variations compared to Figure 1, meaning that the current arousal axis in the Korean structure is more stretched out than that for the Japanese structure.

Locations of the emotion along the social engagement dimension share observable commonalities with Japanese data. Socially disengaging emotions such as “nervous,” and “surprised” were around the top, while socially engaging emotions such as “love,” “delighted,” and “sad” were at the lower end. Important to note, however, that the locations of anger-related emotions (angry, rage, and resentment) do not correspond to the characteristic of this dimension. Anger is generally classified as a socially disengaging emotion (Kitayama et al., 2000, 2006; Kgantsi et al., 2015), but those are placed on the socially engaging side. This may relate to Korean’s indigenous psychology, which is described in the discussion.

Contrasting to the Japanese data, none of the dimensions appear to be clearly distinguishing central and peripheral concepts in Korean structure. Though, it can be said that the social engagement is most likely to be associated with the central/peripheral distinction as nine out of fifteen central emotions were located at the lower half of this dimension and some peripheral emotions (bored and impatient) showed noticeably high level of disengagement. The visual examination of the figures, however, only provides a rough idea on whether the location of central and peripheral concepts differ along each dimension. In order to statistically confirm if the distribution of the 15 central and 15 peripheral concepts along each of the three dimensions differ from each other, the Levene’s Test for Equality of Variances was performed on the MDS coordinates. The results for the Japanese were as follows: valence, *t* (28) = −0.70, *n.s*.; social engagement, *t* (28) = 4.87, *p* < 0.001; arousal, *t* (28) = −2.48, *p* < 0.05. For the Japanese, the distribution of the central and peripheral concepts differed for the social engagement and the arousal dimension, but the effect was much more robust for the social engagement dimension. It confirms the representation in Figure 1 that, for the social engagement dimension, more central concepts are on the socially engaging side and majority of the peripheral concepts are at the socially disengaging side. In terms of arousal, Japanese central concepts tend to be low on that characteristic. The analyses for the Korean dimensions showed no significant results: valence, *t* (28) = 1.52; social engagement, *t* (28) = 1.61; arousal, *t* (28) = −0.88.

Next, the individual dimension weights for Japanese and Korean participants were examined. In the weighted Euclidean analysis, the salience of each dimension for each participant was represented as dimension weights. The more salient the dimension is the more likely people use the relevant characteristics to differentiate emotions. The mean weights for the three dimensions were as follows: Japanese, valence = 0.57, social engagement = 0.28, arousal = 0.25; Koreans, valence = 0.58, social engagement = 0.27, arousal = 0.26. How the weights for the three dimensions differ within each culture was investigated using a within-subject Analysis of Variance (ANOVA) with an effect of dimension. The effect was significant for the Japanese, *F* (2, 124) = 519.47, *p* < 0.001, $\eta_{p}^{2}$ = 0.89, showing that the valence dimension was more salient than the other two, and the social engagement was more salient than the arousal. The valence was also the most salient for the Korean participants, but the weights for the other two dimensions did not differ, *F* (2, 126) = 640.94, *p* < 0.001, $\eta_{p}^{2}$ = 0.91. When the weights for each dimension were contrasted between the two cultures, the level of salience for the valence dimension was equivalent for the Japanese and Koreans, *F* < 1. For the social engagement dimension, however, the weights for Japanese participants were significantly higher than that for the Koreans, *F* (1, 125) = 3.96, *p* = 0.049, $\eta_{p}^{2}$ = 0.03. The result was reversed for the arousal dimension, showing that this dimension was more salient for the Korean than the Japanese, *F* (1, 125) = 4.01, *p* = 0.047, $\eta_{p}^{2}$ = 0.03.

## Discussion

### The Multi-Dimensional Structure of Emotion for Japanese and Koreans

The present study investigated the multi-dimensional structure of emotional concepts among Japanese and Korean individuals by asking them to classify central and peripheral concepts into groups based on similarity. The structure for both cultural groups was represented in the three-dimensional space with valence, social engagement, and arousal. Finding the valence and arousal dimension is consistent with several cross-cultural studies (Russell et al., 1989; Fontaine et al., 2002; Västfjäll et al., 2002; Yik and Russell, 2003).

The present results show that the dimension of valence is culturally universal and the most fundamental property of emotion, as suggested by the core affect theory of emotion (Russell and Barrett, 1999; Russell, 2003; Barrett, 2006). The examination of individual dimension weights supports this account showing that the valence is much more frequently used to distinguish different emotions than the other two characteristics by Japanese and Koreans. Also, the weights to the social engagement and the arousal differed between the two groups of participants. The present study demonstrated that even the arousal dimension, found in many different countries, is heavily influenced by individual variations.

In addition to the frequently observed valence and arousal dimensions, the present research identified the social engagement as an important property. Kgantsi et al. (2015) investigated the level of social engagement for 55 emotions among black and white South African university students. The position of those emotions in their MDS results were similar to those in the present study. This dimension shares some characteristics with the dimension of interactivity (Batliner et al., 2005; Toivonen et al., 2012) and of self-other focused emotion (Markus and Kitayama, 1991). The reason this dimension was identified in the present study is likely to be the collectivistic nature of the samples.

Comparing the self-concepts of individualistic and collectivistic people, the former predominantly consists of self-features that are definable independent from the person’s social relationship (e.g., personal goals, personal beliefs, and personal achievements). The latter also consists of those independent aspects, but the interdependent aspects, which are only definable through the person’s relations with others such as roles and obligations as a group member, are regarded as more important (Markus and Kitayama, 1991). Therefore, collectivistic people might be more sensitive to the distinction of social engagement than individualistic people. Interestingly, it is reported that collectivistic people alter their behaviors when they do not need to consider others, while behaviors of individualistic people are more consistent regardless of the presence of others (Cohen and Gunz, 2002; Uskul and Kikutani, 2014). This indicates that collectivistic people readily distinguish the socially engaging and disengaging situations to choose their behaviors, so it is reasonable to assume that the same distinction is applied to categorization of emotions.

Considering the feature of the social engagement dimension is influenced by culture, it can be said that the dimensions of valence and arousal are culturally universal but other extra dimensions can be culturally specific. Further research involving varieties of cultures is necessary to assess this hypothesis.

### The Social Engagement Dimension and Diversity of Emotion Processing Within Asian Collectivism

Although the presence of social engagement dimension in the present study is likely to be related to the collectivistic nature of the participants, the dimension has been found among individualistic people as well (Kitayama et al., 2000, 2006; Kgantsi et al., 2015), and there seems to be a consensus on which emotions are socially engaging or disengaging. In the present study, the MDS position of the most words were consistent with the characteristics of this dimension, but some emotions violated the expectation. The first example of such emotion is ashamed, which is generally classified as socially engaging emotions, but located at the opposite end in the Japanese and Korean data. Although shame is elicited only in socially engaging situations, the behaviors associated with the emotion tend to be self-isolation. It is because people are motivated to cease further interactions with others to prevent themselves from feeling more shame. This tendency is contrasting to the behaviors triggered by other socially engaging emotions, which predominantly work to increase interpersonal interactions. On a similar note, experience of shame may enhance independent sense of self because one may need to dissociate himself/herself from the social group in which the shame is experienced instead of overcoming the shame to continue belonging to the same group. Shame is a very difficult emotion to define, and some researchers even propose that it shouldn’t be used as a technical term (Kollareth et al., 2019). Also, as a self-conscious emotion, shame can be differently experienced among cultures, since the motives and consequences of shame are attributed to self in variety of ways according to specific socio-cultural value systems (Hong, 2008). Thus, the location of shame-related emotions along the social engagement dimension is likely to vary across cultures.

Similarly, anger-related Korean emotions (angry, rage, and resentment) were not placed as expected. Anger is often experienced when one’s sense of autonomy is threatened by others (Smith and Ellsworth, 1985; Kitayama et al., 2000) and its associated behaviors (e.g., treating others coldly or aggressively) work to push others away (Clark et al., 1996; Van Kleef et al., 2010). Thus, anger is often regarded as a socially disengaging emotion (Rothman and Magee, 2016). However, “angry,” “hate” and “resentment” in the present Korean data showed that they are socially engaging.

Korean resentment, *han*, is thought to be a culturally unique emotion and definition of this emotion might provide some clues on why Korean anger-related emotions are socially engaging. *Han* is a very complex emotion, representing helplessness, ego lamentation and grief caused by frustration and loss. This emotion also represents some positive aspects such as hope for future enjoyment and for divine lights. Thus, it is distinguished from the emotion of resentment in English, which is governed by a sense of bitterness and motivation for revenge (Chon, 1993). *Han* has a root in Korean war history, mainly based on their experience of defeats by neighboring big powers such as China and Japan. Therefore, it is related to anger and hate, but also consists of positive connotations such as appreciation for the present or hope for the better future. Importantly, those connotations of *han* are, precisely speaking, not about experience of individuals but about experience of the nation (Ko, 1980). The emotion is passed on from older generations to younger generations along with stories of wartime experiences, which often involves losing touch with family members. The sharing of *han*, therefore, cultivates the sense of national unity and cultural belongings among Koreans (Lee and Choi, 2003). This makes *han* a socially engaging emotion. Although “angry” and “rage” in Korean are not regarded as culturally specific, they are nonetheless related to *han* and thus, they might also be judged as socially engaging.

The emotion of “like” and “lonely” in Korean were also placed at unexpected places. These emotions are regarded as socially engaging, and it was the case for the Japanese data. For “like,” according to Japanese and Korean dictionaries, the Japanese word has identical meanings to the English word, but the meaning of the Korean word is somewhat different. Japanese and English “like” can often be used interchangeably with “love” because the like, similar to “love,” tend to imply one’s emotional investment in the relationship between him/her and the liked objects or humans. Thus, the like experience for Japanese and English fosters the sense of interdependent self. In Korean, “like” and “love” are rarely used interchangeably because “like” experience is directed more toward self than the relationship, while “love” is regarded as a distinct relation-focused experience. The like experience for Koreans is thought to help them understanding and defining themselves clearly (e.g., “I understand myself as a person who likes this sport and that food”) and so, it may function as a socially disengaging emotion. This might be the reason why Korean “like” was categorized as socially disengaging. The word lonely indicates one’s lack of connection and psychological desires/needs for it. At this moment, it is not clear why Korean “lonely” was judged as socially disengaging, but it might be the case that it emphasizes the status of being disconnected from others. Korean has many variations of lonely and each has slightly different connotations and so, it is possible that the meaning of the word used in the present research did not match the meaning of the Japanese or English word.

These examples of emotions which were not located at the expected place indicate that the socially engaging/disengaging categorization is rather flexible. It is because the categorization criteria based on English meaning of emotion words do not always apply to the supposedly equivalent words in other languages. As the word “like” in the present research, the native words in Japanese and Koreans mean something quite different from each other but the equivalent English words was the same for both. This is a fundamental problem for research that compares linguistic cognition of different cultures, because any difference in cognitive processing is inherently influenced by language variations (Whorf, 1956). This issue is further discussed in the limitation section. As mentioned before, the dimension other than valence and arousal can be culturally variable and thus, locations of emotions along such dimension strongly reflect culturally specific interpretation of emotion words. More research is necessary to further investigate the characteristics of the social engagement dimension and how each emotion is categorized.

Nevertheless, the present results suggest that the social engagement dimension is associated with the distinction of central and peripheral concepts for the Japanese and Korean people, albeit the tendency was weak among Koreans. Both groups of participants regarded socially engaging emotions as more typical examples of emotions. This likely to reflect their collectivistic nature which prioritize group harmony (Caudill and Weinstein, 1969; Sue, 1973; Leong, 1992; Uba, 1994). Kitayama and Markus (2000) demonstrated that individualistic people experience socially disengaging emotions more frequently and intensely than collectivistic people, while socially engaging emotions were more frequently and intensely experienced by collectivistic people. This suggests that the collectivistic individuals prioritize socially engaging emotional experiences and, consequently, regard them as more typical examples of emotion. Some researchers also suggest that culturally unprioritized emotions are less frequently experienced because they are consciously suppressed or regulated (De Leersnyder et al., 2013), thus those emotions might be deliberately categorized as less important (peripheral).

The central/peripheral distinction along the social engagement dimension was much less clear for the Korean than for the Japanese. Park et al. (2018) compared the distinction between Japanese and Korean participants and found that Koreans do not distinguish these two types of concepts as readily as Japanese. Thus, it is likely that the distinction is much less salient for Koreans. Another possibility is a difference in self-construal during interpersonal relationships between Japanese and Koreans. Inumiya (2009) reports that while Koreans tend to regard themselves as a center of the relationship who exerts influence on others, Japanese viewed themselves as a marginal entity that accepts social influence. Hur (2015) also points out that, although both Japanese and Korean are collectivistic, the focused relationship is the group for the former and individual others for the latter. This means that, on the one hand, Japanese focus on their status, role, and whether they are fitting in a particular social group to define themselves. On the other hand, Koreans focus on their relationship with each of the others surrounding them, and how they perform in each relationship is a crucial part of their self-concept. For example, in a relationship at work, Japanese would view their boss and colleague as the members of the same social group. So, the important aspect of the Japanese person’s self-concept would be how well he/she performs as a group member rather than how he/she performs in the separate relationship with the boss or with the colleague. For Koreans, on the contrary, their relationship with their boss and colleague are both independently important for their self-concept. Combining this account of Hur (2015) and the forementioned Inumiya’s finding (Inumiya, 2009), it can be suggested that the crucial component of Korean self-concept is how individuals exert influence on each of the others in relationships. Han et al. (2009) summarize that the important component of self-concept for Japanese is objectivity and that for Korean is subjectivity. Among Koreans, socially disengaging emotions, say, pride, can be experienced within a social context in which they are proud of themselves with their influence on another person. This tendency may refrain Koreans from regarding socially disengaging emotions as less important than socially engaging ones, resulting in the vague distinction between the central and peripheral concepts.

### The Arousal Dimension for Koreans

The present results revealed that the range of arousal dimension is different between the Japanese and Korean structures. The results suggest that the arousal level of Korean emotions is generally high due to the lack of extremely low arousal emotions. This resulted in the Korean conceptual space in Figure 2 to cover a narrower range of arousal variation than the Japanese structure, looking like the vertical axis of this figure is more stretched than the Japanese space. The absence of words with extremely low arousal levels can completely obscure the presence of this dimension sometimes (Ahn et al., 1993; Lee and Ahn, 1997). Therefore, the question is why the 15 peripheral concepts in the present study, which were randomly chosen from a larger set of words in Park et al. (2018), lacked those words. It could be just a chance that they were not selected, but it is more likely that low-arousal concepts are not as common as those of high-arousal in Korean. Kim and Ahn (2011) categorized 140 Korean emotion words into aroused and non-aroused groups and found that only 37.1% were judged as non-aroused. A similar result was found by Park and Min (2005), revealing that only 172 (39.6%) out of 434 Korean emotion words were categorized as non-arousal. Both studies also point out that positive non-arousal emotion words are particularly rare among Korean emotion concepts. These findings are consistent with the notion that Korean emotions mark a generally higher arousal level than emotions in other cultures (Kuppens et al., 2006; Park and Park, 2009; Uchida and Kitayama, 2009). Also, as mentioned in the introduction, Koreans are eager to express honest emotion to others and use communications to understand each other (Lee and Matsumoto, 2011). This implies that emotions are communicative for Koreans, and they may regard extremely low arousal emotions as less urgent in communication than high arousal ones.

The present MDS results suggest that Koreans allocate wider cognitive space for arousal dimension compared to Japanese and this means they evaluate this characteristic much more finely than Japanese do. This may relate to communicative nature of Korean people, who require suitable words for varieties of communication needs. This is similar to Eskimo languages having vastly more numbers of snow-related words than English (Boas, 1911) due to people’s need for precise descriptions of their environment (Whorf, 1956). In comparison, Japanese are not as expressive in terms of arousal, having weaker needs for word variations for communication (Lee, 2018).

The Korean indigenous emotion of *Shimcheong* (or often spelled as *Simjeong*) also explains why Koreans are arousal focused. *Shimcheong* is an aroused affective state elicited by the interactions of two or more people and how this matches expectation. In any social interactions with known others, we have certain expectancy toward the others’ behaviors. As long as others’ behavior falls into one’s expectation, he/she would not experience *Shimcheong*, but once the behaviors of others violate the expectation *Shimcheong* occurs like a ripple on a calm lake. *Shimcheong* then elicits further processing of the situation and leads the person to experience more detailed and valenced emotions such as happiness or anger depending on whether the expectation is violated in a positive or negative way (Choi et al., 2007). According to Choi and Kim (1999), Koreans believe that individuals’ internal states rather than observable behaviors reflect the person’s true self, thus his/her internal state is always subject to social evaluations. *Shimcheong*, therefore, also works in private situations because Koreans internalize other people’s points of view and assess whether their own behaviors are within others’ expectations. This indigenous emotion suggests that Koreans are very sensitive to the state of arousal.

### Limitations

The present study has some limitations, and using unmatched word sets for the Japanese and Koreans can be regarded as a major shortcoming in cross-cultural research. More objective comparisons across countries can be achieved using a matched set of emotion words. Also, considering that even equivalent words in two languages can contain some nuanced differences as “like” in the present research, the more controlled investigation can be done with bilinguals. For example, Japanese-English and Korean-English bilinguals perform the word grouping task in English. Having said that, using culturally specific emotions contributed to deepening our understanding of how culture influences emotional experiences.

Unbalanced number of men and women in the sample (there were more than twice as many women as men) was also a shortcoming of the present research. The gender difference in emotion experience is well documented although the findings are often inconsistent depending on participants’ internal traits, situational variables including social and cultural influences, types of emotional processes examined, and experimental tasks used (see Brody and Hall (2008), for review). Therefore, the present results may consist of some gender bias. Furthermore, the sample size of the present research is not large enough to support a split MDS analysis for each gender, thus the presence of such bias cannot be investigated. However, the task in the study was solvable by simply using semantic definitions of the words and therefore, the task was relatively independent from situational or personal factors such as gender stereotypes, ability of emotional regulations, and sensitivity to emotional cues, for which gender differences are often reported (Brody and Hall, 2008). Nevertheless, similar study should be conducted with a more balanced sample in the future for the better generalization of the present findings.

## Conclusion

The present study compared the multi-dimensional structure of emotion concepts between Japanese and Korean individuals. The results revealed a common three-dimensional structure, but the importance among those dimensions was different between the two groups. The relatively novel dimension of the social engagement may be identified because the present samples were collectivistic East Asians, and this suggests a strong possibility that the dimension other than valence and arousal is culturally variable. Emotion concepts reflect how people understand self and others, and these aspects may be diverse even within collectivistic cultures. For example, Choi (1993) reported that Japanese and Koreans regard others very differently by focusing on the concept of “us” and “in-group affection.” Korean “us” is conscious feelings and experiences normally shared among relatives but extended to other in-group members. This means Koreans feel family like strong affection to other group members once they are included in “us.” On the contrary, the Japanese “us” is a sense of collective community and the experience resulting from shared goals and activities. Japanese also express “in-group affection” in the context of joint activities based on collective norms, not in a context-free manner as in family relations. The differences in self-construal during interpersonal relationships, such as focusing on objectivity or subjectivity (Han et al., 2009) are also reported. Such diversity in understanding self and others, which is present within a collectivistic cultural group, is likely to impact how people perceive and express emotions and consequently impact the multi-dimensional structure for emotion concepts.

## Data Availability Statement

The raw data supporting the conclusions of this article will be made available by the authors, without undue reservation.

## Ethics Statement

The studies involving human participants were reviewed and approved by the Ethical Committee of Doshisha University. The patients/participants provided their written informed consent to participate in this study.

## Author Contributions

E-JP was involved in the experimental design, data collection, data analysis, interpretation, and manuscript writing. MK was involved in the data analysis, interpretations, and manuscript writing. MI was involved in the data analysis and interpretation work. J-HL was involved in the experimental design and he oversaw the data collection in South Korea. NS was involved in the experimental design, data analysis, and interpretation work. All authors contributed to the article and approved the submitted version.

## Conflict of Interest

The authors declare that the research was conducted in the absence of any commercial or financial relationships that could be construed as a potential conflict of interest.

## Publisher’s Note

All claims expressed in this article are solely those of the authors and do not necessarily represent those of their affiliated organizations, or those of the publisher, the editors and the reviewers. Any product that may be evaluated in this article, or claim that may be made by its manufacturer, is not guaranteed or endorsed by the publisher.
