# Supplementary material for: Properties of Central and Peripheral Concepts of Emotion in Japanese and Korean: An Examination Using a Multi-Dimensional Model
Source: Front Psychol. 2022 Feb 15;13:825404. doi: 10.3389/fpsyg.2022.825404 (PMC8885600; doi:10.3389/fpsyg.2022.825404)
Supplement: Supplementary file 1 [file Data_Sheet_1.docx]

##
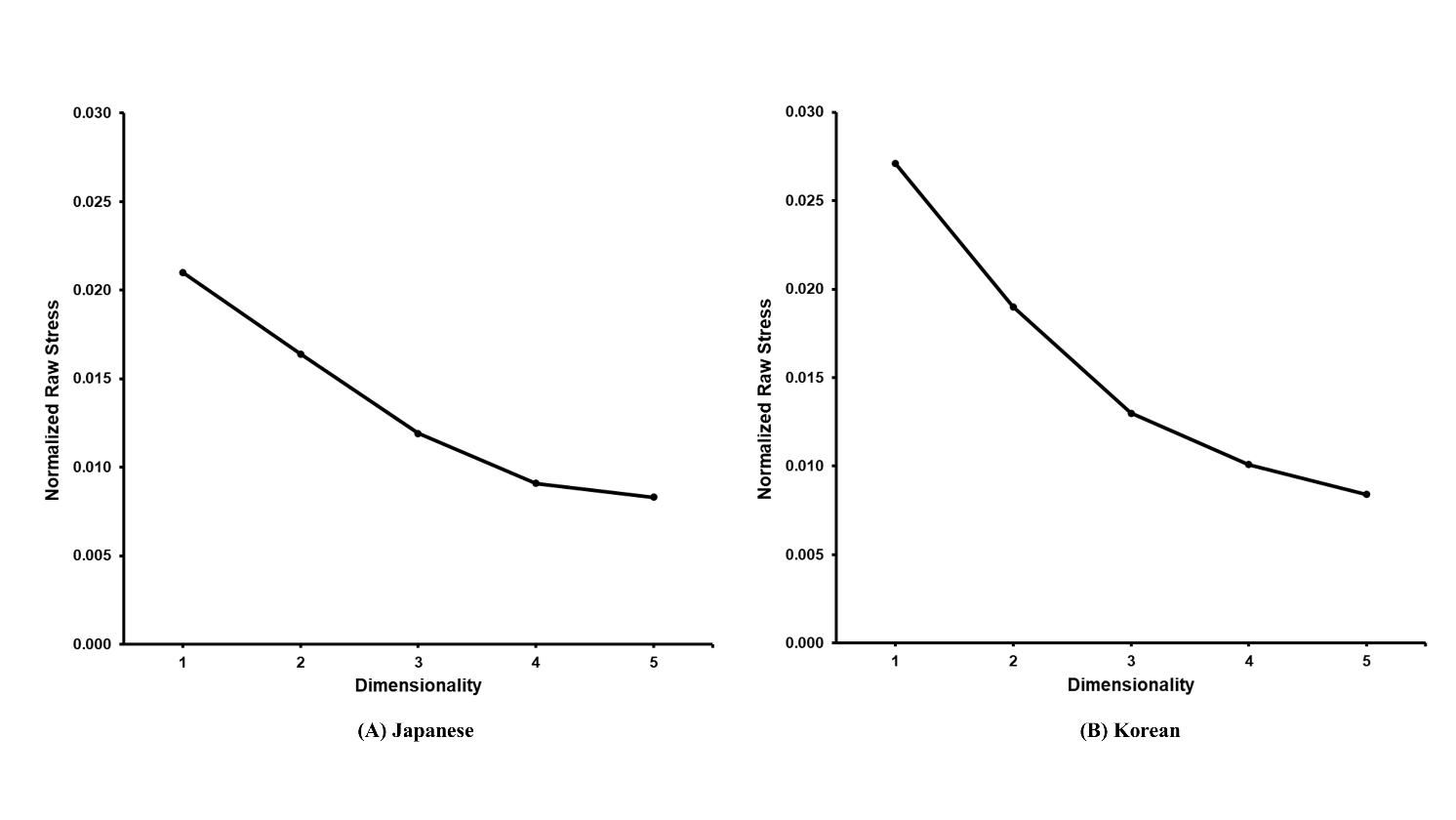


**Supplementary Figure 1.** Scree plot displaying an elbow at three dimensions in Japanese and Korean
